# Response to: Comment on “Effective Range of Percutaneous Posterior Full-Endoscopic Paramedian Cervical Disc Herniation Discectomy and Indications for Patient Selection”

**DOI:** 10.1155/2020/6387143

**Published:** 2020-04-23

**Authors:** Hongquan Wen, Xin Wang, Wenbo Liao, Weijun Kong, Jianpu Qin, Xing Chen, Hai Lv, Thor Friis

**Affiliations:** ^1^Department of Orthopaedic Surgery, Affiliated Hospital of Zunyi Medical University, Zunyi, 563000 Guizhou, China; ^2^Department of Orthopaedic Surgery, Affiliated Hospital of Shaanxi University of Traditional Chinese Medicine, Xianyang, 712000 Shaanxi, China; ^3^Institute of Health and Biomedical Innovation, Queensland University of Technology, Brisbane, 4059 Queensland, Australia; ^4^Academy of Orthopedics of Guangdong Province, Department of Orthopedic Surgery, The Third Affiliated Hospital of Southern Medical University, Guangzhou, 510630 Guangdong, China

We thank Jun-Song Yang et al. for highlighting some important issues [1] in our study, “Effective Range of Percutaneous Posterior Full-Endoscopic Paramedian Cervical Disc Herniation Discectomy and Indications for Patient Selection” [2].

For the patients accompanying with huge paramedian cervical disc herniation, who were not included in our study, open surgery was more suitable because the characteristics of full-endoscopic operation are accurate decompression and targeted extraction. The scope of endoscopic decompression was, therefore, limited. There may be incomplete decompression and dissatisfaction with the recovery of symptoms of the patients with huge paramedian cervical disc herniation.

The objective of this study was to explore the effective range of percutaneous posterior full-endoscopic paramedian cervical disc herniation discectomy. The medial margin of uncovertebral joint is generally not exposed under endoscopic operation, which is not instructive for us to measure the safe and effective range of the resectable herniated disc.

Jun-Song Yang et al. asked whether T1-weighted MRI may be more appropriate to locate the medial border of discectomy at the early stage postoperatively. For the T1-weighted MRI and T2-weighted MRI, cerebrospinal fluid and residual fluid are low signal and high signal, respectively. The spinal cord is low or equal signal in T2-weighted MRI. The border of the spinal cord, dural sac, and cerebrospinal fluid is, therefore, clearer in T2-weighted MRI than in T1-weighted MRI.

Jun-Song Yang et al. pointed out that in the postoperative follow-up, the distance between the edge of the dural sac and the inside edge of the intervertebral disc was significantly smaller than between the edge of the dural sac and the inside edge of the herniated disc. The article should have stated that postoperative DSMD is less than the preoperative DSMHD, and we agree with this statement because it is more detailed and appropriate.

We had explained the retraction of the protruding nucleus pulposus after the intradiscal decompression, such as the resected amount of actual intervertebral disc tissues was less than that of the preoperative measurements, which had been shown in MRI. This, however, was not the major reason in the process of the improvement of clinical outcome. Relieving the compression of the spinal cord and nerve root was critical to the improvement of clinical outcome after the protruding disc was resected.

After the foraminal unroofing and the resection of the ligamentum flavum, excessive traction can cause damage to the spinal cord and nerve root and the range of accommodation and movement is limited. The spinal canal was not large enough to accommodate the endoscope and was not available for the spinal cord and nerve root to compensate the compression from the ventral protruded nucleus pulposus.
